# Factors associated with pericardial effusion development and persistence in systemic sclerosis: a single-centre retrospective analysis

**DOI:** 10.1007/s00296-025-05925-0

**Published:** 2025-07-14

**Authors:** Nikolaos Koletsos, Evripidis Kaltsonoudis, Eleftherios Pelechas, Alexandros A. Drosos, Paraskevi V. Voulgari

**Affiliations:** Department of Rheumatology, School of Health Sciences, Faculty of Medicine, University of Ioannina, Stavrou Niarchou Avenue, 45500 Ioannina, Greece

**Keywords:** Systemic sclerosis, Pericardial effusion, Pulmonary hypertension, Interstitial lung disease, Risk factors

## Abstract

Heart involvement in Systemic Sclerosis (SSc) remains among the leading causes of disease-related deaths. SSc has been associated with an increased risk of pericardial effusion (PerEff). The aim of our study was to examine the prevalence and identify possible factors associated with ever and persistent SSc-PerEff. For this retrospective study, consecutive patients with a diagnosis of SSc, that visited our Outpatient Unit until June 2024, were recruited. Demographic data, disease manifestations, serological profile, and internal organ involvement were collected. Initially, participants with at least one measurement of PerEff were enrolled. For the second step individuals with at least two echocardiograms were included and were classified into three groups: single, persistent and never PerEff. In total 290 adult patients (female-to-male ratio 5.6:1) were included. Results showed that 24.5% of the patients had at least one echocardiogram ever positive for PerEff and 13.2% exhibited persistent PerEff. In the multivariate analysis, ever PerEff was associated with the presence of pulmonary hypertension [PH, Odds ratio, (OR):3.22, *p* < 0.01], interstitial lung disease (ILD, OR:4.18, *p* < 0.01), telangiectasias (OR:4.25, *p* < 0.01) and elevated erythrocyte sedimentation rate (ESR, OR:2.01, *p* < 0.05). Persistent PerEff was, also, associated with PH (OR:2.90, *p* < 0.05), ILD (OR:6.65, *p* < 0.05), telangiectasias (OR:4.84, *P* < 0.05) and elevated ESR (OR:2.76, *p* < 0.05). PerEff is a common disease-related manifestation. Both ever and persistent PerEff were associated with PH, ILD, telangiectasias and increased inflammatory markers. The present study further supports the clinical significance of PerEff in SSc patients, with important implications in disease course and management.

**Supplementary Information:**

The online version contains supplementary material available at 10.1007/s00296-025-05925-0.

## Introduction

Systemic sclerosis (SSc) is a chronic and progressive autoimmune disease with a female predominance [1, 2]. It is associated with significant complications that affect both survival and quality of life [3, 4]. More specifically, in an analysis using studies after 1990, cumulative 5-years survival from disease diagnosis was calculated to be almost 85% [5]. The presence of significant complications, however, such as interstitial lung disease (ILD) or pulmonary hypertension (PH) and especially their coexistence seems to be related with worse survival rates [6, 7].

The heart is a frequent and major organ involvement during the disease course, remaining among the leading causes of SSc-related deaths [8–10]. Cardiac involvement can affect all the heart structural components and has been associated with several risk factors including demographics (e.g. male sex and age), clinical manifestations (e.g. swollen joints or telangiectasias) and positive anti-topoisomerase I (Scl-70) antibodies [8, 11, 12]. Pericardial effusion (PerEff) is a known complication of SSc that has been reported even from the early studies [13]. It can be detected up to 41% by echocardiography and the percentage is even greater (up to almost 80%) in necrotomic studies [14–16]. More recent data suggest that SSc is associated with a 6x-fold increased risk of developing pericarditis [17]. PerEff in SSc is usually asymptomatic, but it can also be the presenting manifestation [8, 16, 18, 19]. In general, clinical presentation of PerEff varies according to the speed of pericardial fluid accumulation and the etiology of the effusion [20, 21]. Regarding presentation, clinically symptomatic PerEff in SSc does not differ from PerEff by other causes, with the most common symptoms being shortness of breath and chest pain [22, 23]. Rarely (less than 1% of hospitalised SSc patients) cardiac tamponade can be identified and it is associated with increased mortality compared to PerEff without a tamponade physiology [24]. Pericardial involvement may be more frequent in SSc patients with diffuse skin subtype as compared to both limited skin subtype and SSc sine scleroderma [25].

PerEff is known to be associated with PH and the presence of PerEff in patients with pulmonary arterial hypertension has been associated with increased mortality [26]. Those findings have been confirmed in patients with SSc as well. Data from the PHAROS registry showed that half of SSc patients with PH have at least one echocardiogram positive for PerEff at or after PH diagnosis [14]. Furthermore, the presence of PerEff has been associated with increased risk for hospitalisation and mortality in SSc patients at risk for PH while, more recently, persistent PerEff has been associated with increased mortality in SSc patients with PH [14, 27].

However, apart from PH, there is limited data regarding the associations of PerEff with other disease related characteristics (including demographics, clinical manifestations and organ involvement, antibodies and inflammatory markers). Therefore, the aim of our study was to (i) examine the prevalence of ever and persistent PerEff and (ii) try to identify possible factors that could be associated with ever and persistent PerEff in a homogeneous cohort of SSc patients from a single tertiary center.

## Materials and methods

### Study design and participants

This is a single center, retrospective, observational study. Consecutive, adult (> 18 years old) patients with a definite diagnosis of SSc, that visited the Outpatient Unit of the Rheumatology Clinic, of the University of Ioannina over the last 30 years (until June 2024) were recruited. The diagnosis of SSc was made by a rheumatology specialist and the date of diagnosis was defined from the time of the first non-Raynaud symptom or sign. Patients were categorised as having diffuse or limited disease and cases of localized scleroderma were excluded [28]. All individuals fulfilled the most recent classification criteria at the time of the diagnosis [29, 30]. The study was approved by Institutional Review Board Committee of the University Hospital of Ioannina, Greece (protocol number 6984, date of approval March 11th, 2024) and was conducted in accordance with the Declaration of Helsinki [31]. Patient consent was not required for this retrospective study and no identifiable patient information was recorded.

### Data collection

Data were derived from patients’ medical records until the last registered visit. Information included: baseline sociodemographic characteristics, type of skin involvement, digital ulcers, telangiectasias, arthritis, gastrointestinal (GI) tract involvement [including symptoms (i.e. reflux, dysphagia, bloating) or signs of GI involvement in endoscopy/esophageal manometry or imaging techniques (i.e. hypomotility or dilation of the esophagus)] and medication history. Regarding pulmonary involvement, ILD was confirmed by chest radiograph and/or HRCT scan, at any time from diagnosis to the last clinical assessment. In addition, pulmonary function tests nearest to the last clinical evaluation were reviewed and forced vital capacity (FVC) along with carbon monoxide diffusing capacity (DLCO), adjusted for haemoglobin levels, were recorded. The presence of pulmonary hypertension [determined by transthoracic echocardiography (TTE) or right heart catheterization] and heart involvement [including estimated ejection fraction, presence of PerEff and diastolic dysfunction, assessed by TTE] were also recorded. All TTEs were carried out by specialized cardiologists, only, according to the current practice guidelines at the time they were performed. The immunological profile included antinuclear antibodies (ANA), anti-topoisomerase I antibodies (Scl-70), anti-centromere antibodies (ACA) and anti-Ro antibodies. Elevated inflammatory markers (Erythrocyte Sedimentation Rate, ESR > 30 mm and C-reactive protein, CRP over normal values) were also recorded, as described elsewhere [32, 33].

For the analysis a two-step approach was applied. Therefore, only participants with at least one measurement of PerEff by echocardiogram were initially enrolled. When investigating characteristics of patients with persistent PerEff, only individuals with at least two echocardiograms were included in the analysis. For the second step, individuals were classified into three groups: (i) Single PerEff, defined as having only one echocardiogram positive for pericardial effusion, (ii) Persistent PerEff defined as having at least two echocardiograms positive for PerEff (either consecutive or intermittent) and (iii) never PerEff, defined as the absence of pericardial effusion in all available echocardiograms.

### Statistical analysis

Statistical analysis was performed using SPSS software (IBM SPSS Statistics 25.0, Chicago, IL, USA). Normally distributed continuous variables are described as mean ± standard deviation, while non-normally distributed as median ± interquartile range, based on the normality of the distribution. Differences between groups were examined by independent samples t-tests or one-way ANOVA for normally distributed variables, whereas the non-parametric Mann-Whitney or Kruskall-Wallis was used for non-normally distributed variables. Tukey post-hoc analysis was used for pairwise analysis, in case of a significant ANOVA. Qualitative variables were compared using the chi-square test, with Fisher’s exact test when necessary, or Phi and Cramer’s V, for variables with more than two groups and results are expressed as percentages.

Multivariate logistic regression analyses were applied to determine predictors of both ever and persistent PerEff. Factors known or expected to be associated with the investigated dependent variable (such as age, male sex, the presence of PH or ILD, diffuse skin subtype, anti-Scl-70 positivity and increased inflammatory markers) were tested as regressors in univariate analysis. Based on the results of the univariate analyses, independent variables with a p value < 0.2 were included in the multivariate regression models, to adjust for potential confounders. Backward elimination was implemented, to refine the multivariate model and odds ratios (OR) with their 95% confidence intervals (CIs) were calculated to quantify the associations. A p value < 0.05 was considered statistically significant in all tests.

## Results

### Ever pericardial effusion

A total of 290 SSc patients were included. All participants were of Caucasian origin. The female-to-male ratio was 5.6:1 and 71.0% had limited SSc. Mean age of the population at inclusion was 59.8 ± 14.4 years and median disease duration was 8.0 [interquartile range (IQR) 2.6–15.2] years. Among the initial population, 24.5% had at least one echocardiogram positive for PerEff. The majority of the cases were asymptomatic. Only one patient developed cardiac tamponade and was transferred to the cardiac intensive care unit, where a successful pericardiocentesis was performed. None of the participants had symptoms or recent infection.

Demographic and clinical characteristics of the two groups (ever-never PerEff) are presented in Table 1. Individuals with ever PerEff had older age at diagnosis (52.2 ± 13.1 vs. 48.1 ± 15.2 respectively, *p* < 0.05). A greater proportion of patients who ever had PerEff, had GI involvement (*p* < 0.05) and telangiectasias (*p* < 0.001). Moreover, ILD and PH were significantly more frequent among individuals who ever had PerEff (*p* < 0.001 for both comparisons). Additionally, patients who ever had PerEff exhibited significantly lower FVC [77.0 (28.0) vs. 92.4 (24.0), *p* < 0.001], lower DLCO [55.5 (26.0) vs. 73.3 (32.0), *p* < 0.001] and higher systolic Pulmonary Artery Pressure [sPAP 38.0 (30.0) vs. 30.1 (11.5), *p* < 0.001] values as compared to those who never had PerEff. Individuals with ever PerEff presented more frequently increased ESR (*p* < 0.01) and a trend towards more CRP elevation (*p* = 0.05). No statistically significant differences were observed regarding skin subtype or other clinical characteristics.

**Table 1:** Baseline characteristics and manifestations of SSc patients that ever or never had PE

	Ever PE (*n* = 71)	Never PE (*n* = 219)	*P* value
Age (years), mean ± S.D.	63.9 ± 12.1	58.4 ± 14.9	**0.003**
Age of diagnosis (years), mean ± S.D.	52.2 ± 13.1	48.1 ± 15.2	**0.047**
Disease duration (years), median (IQR)	9.3 (10.7)	8.0 (13.1)	0.152
Ever smoker, yes (%)	26.8	34.1	0.251
Female sex, (%)	88.7	83.6	0.291
Diffuse SSc, yes (%)	36.6	26.5	0.102
Limited SSc, yes (%)	63.4	73.5	
Raynaud’s phenomenon	98.6	96.3	0.693
History of digital ulcers, (%)	62.0	51.6	0.127
Telangiectasias, yes (%)	84.5	61.5	**< 0.001**
GI involvement, yes (%)	73.2	59.8	**0.042**
Arthritis, yes (%)	36.6	32.9	0.562
ILD presence, (%)	80.0	55.7	**< 0.001**
FVC (%), median (IQR)	77.0 (28.0)	92.4 (24.0)	**< 0.001**
DLCO (%), median (IQR)	55.5 (26.0)	73.3 (32.0)	**< 0.001**
LV EF (%), median (IQR)	65.0 (10.0)	65.0 (10.0)	0.604
sPAP (mmHg), median (IQR)	38.0 (30.0)	30.1 (11.5)	**< 0.001**
LV diastolic dysfunction, (%)	48.5	36.1	0.071
Pulmonary hypertension, (%)	50.0	16.9	**< 0.001**
ANA, yes (%)	97.2	95.8	0.607
ACA, yes (%)	25.4	31.0	0.364
Scl-70, yes (%)	47.9	35.6	0.066
ESR elevation, (%)	44.1	25.7	**0.004**
CRP elevation, (%)	36.2	24.2	0.050
Immunosuppressants, (%)	81.7	72.1	0.109
Corticosteroids, (%)	66.2	53.5	0.060
Vasoactive drugs, (%)	76.8	51.8	**< 0.001**

SSc: Systemic sclerosis; PE: Pericardial effusion; S.D.: Standard deviation; IQR: Interquartile range; GI: Gastrointestinal; ILD: interstitial lung disease; FVC: forced vital capacity; DLCO: carbon monoxide diffusing capacity; sPAP: Systolic pulmonary artery pressure, as assessed by echocardiography; LV: Left ventricle; EF: Ejection fraction; ANA: antinuclear antibodies; ACA: anti-centromere antibodies; Scl-70: anti-topoisomerase I antibodies; ESR: Erythrocyte sedimentation rate; CRP: C-reactive protein

Regarding treatment (Table 1), no differences were detected in total use of immunosuppressants between the two groups. On the other hand, vasoactive drugs were used significantly more often by patients who ever had PerEff (76.8% vs. 51.8% respectively, *p* < 0.001), while a trend for greater corticosteroid use (66.2% vs. 53.5% respectively, *p* = 0.060) was also observed among individuals who ever had PerEff.

**Fig. 1:**
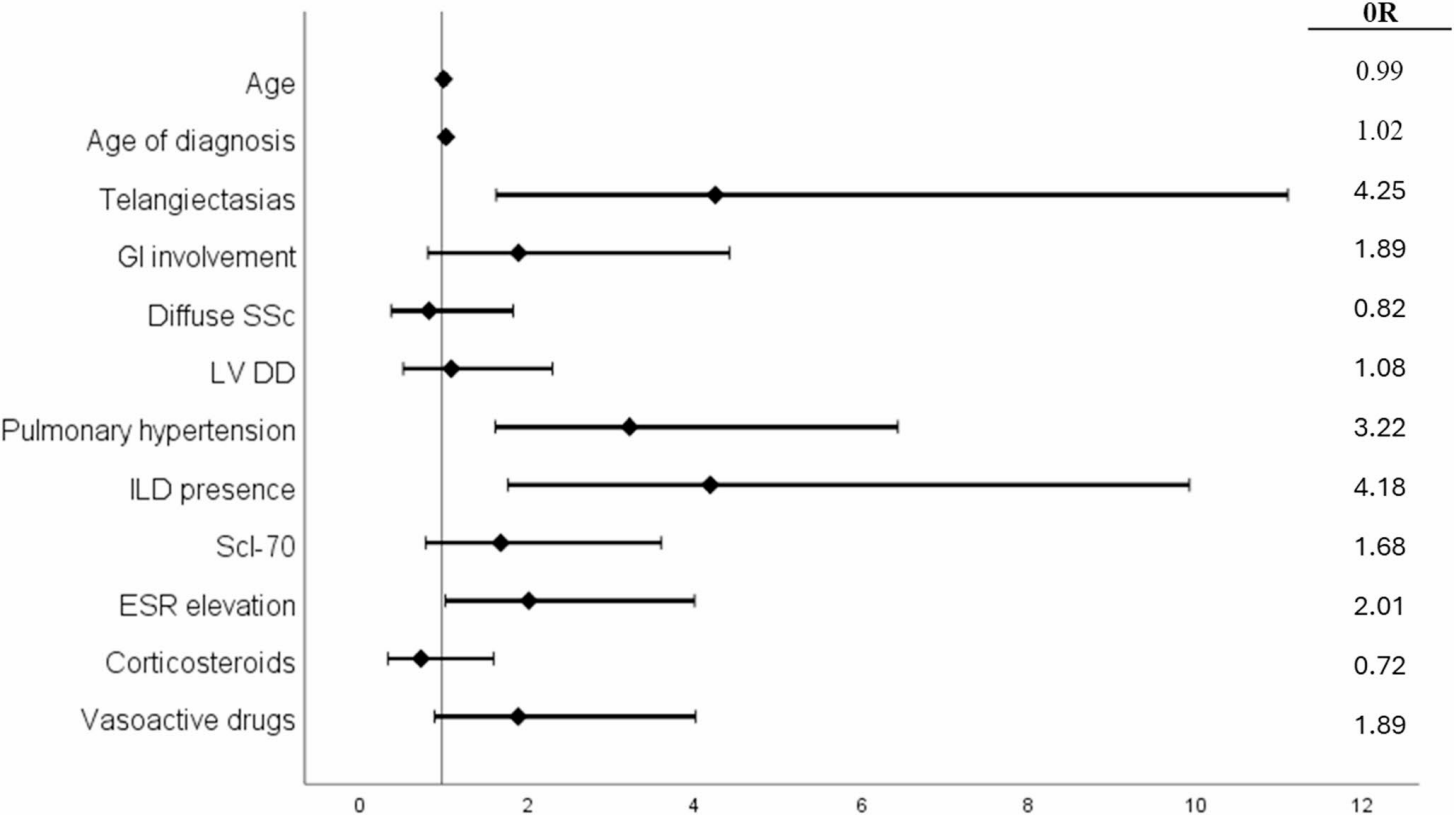
Characteristics associated with ever pericardial effusion in SSc patients in the multivariate logistic regression. GI: Gastrointestinal; SSc: Systemic sclerosis; LV DD: Left ventricle diastolic dysfunction; ILD: interstitial lung disease; Scl-70: anti-topoisomerase I antibodies; ESR: Erythrocyte sedimentation rate

Univariate and multivariate analyses results for ever PerEff are summarised in Table S1 (see supplementary data available). In the univariate analysis a significant association was observed with current age (*p* < 0.01), age of SSc diagnosis (*p* < 0.05), vasoactive drug use (*p* = 0.001), the presence of PH (*p* = 0.001), ILD (*p* = 0.001) and telangiectasias (*p* = 0.001), GI involvement (*p* < 0.05), as well as ESR elevation (*p* < 0.01). In the multivariate analysis the presence of PH [OR 3.219 (95% CI: 1.612–6.430)], ILD [OR 4.184 (95% CI: 1.765–9.917)], telangiectasias [OR 4.245 (95% CI: 1.624–11.095)] and increased ESR [OR 2.013 (95% CI: 1.014–3.997)] remained associated with ever having PerEff (Fig. 1, Table S1). When increased CRP was included as a binary variable in the analysis instead of increased ESR levels, it was excluded from the final model, leaving the rest parameters unaffected. Overall, the model predicted correctly 78.7% of the cases and neither interaction between PH and ILD, nor between PH and vasoactive drugs made a statistically significant contribution to the multivariate model (Table S1, see supplementary data available).

### Persistent pericardial effusion

For the secondary analysis, 70 patients were excluded because they had only one measure of PerEff. A total of 220 SSc patients had at least two echocardiograms during their follow up and were included in the final analysis. Mean age of the population was 60.7 ± 14.0 years and median disease duration was 9.5 [interquartile range (IQR) 4.4–17.0] years.

Among the individuals included in the comparative analysis, 15.9% had a single episode of PerEff, 13.2% had persistent PerEff, while 70.9% were persistently negative for PerEff. Baseline characteristics of the three groups are presented in Table 2. Individuals with never PerEff had significantly lower frequency of PH, ILD and telangiectasias (*P* < 0.01 for all comparisons). Additionally, patients who never had PerEff, exhibited significantly higher FVC, higher DLCO and lower sPAP values (*p* < 0.001 for all comparisons). SSc patients with persistent PerEff had more frequently elevated ESR (*p* < 0.05), while no statistically significant differences between the groups were observed in CRP elevation. Regarding treatment (Table 2), no differences were detected in total use of immunosuppressants whereas vasoactive drugs and corticosteroids were significantly less frequently used by individuals that never had PerEff (*p* < 0.05 for both comparisons).

**Table 2:** Baseline characteristics and manifestations of SSc with persistent, ever or never PE

	Persistent PE (*n* = 29)	Ever PE (*n* = 35)	Never PE (*n* = 156)	*P* value
Age (years), mean ± S.D.	64.1 ± 12.5	64.9 ± 9.6	59.1 ± 14.8	**0.034**
Age of diagnosis (years), mean ± S.D.	52.4 ± 12.6	52.3 ± 13.5	47.3 ± 15.3	0.069
Disease duration (years), median (IQR)	10.7 (10.8)	9.2 (17.1)	9.6 (12.7)	0.995
Ever smoker, yes (%)	34.5	25.7	34.9	0.579
Female sex, (%)	89.7	88.6	83.3	0.554
Diffuse SSc, yes (%)	44.8	34.3	28.8	0.224
Limited SSc, yes (%)	55.2	65.7	71.2	
Raynaud’s phenomenon	100.0	97.1	97.4	0.674
History of digital ulcers, (%)	65.5	60.0	53.8	0.454
Telangiectasias, yes (%)	93.1	82.9	63.9	**0.001**
GI involvement, yes (%)	75.9	77.1	62.2	0.121
Arthritis, yes (%)	41.4	31.4	34.6	0.696
ILD presence, (%)	93.1	77.1	61.5	**0.002**
FVC (%), median (IQR)	78.5 (27.0)	77.0 (24.5)	95.0 (24.5)	**< 0.001**
DLCO (%), median (IQR)	56.3 (24.5)	51.5 (24.4)	72.0 (31.3)	**< 0.001**
LV EF (%), median (IQR)	62.4 (6.8)	65.0 (10.0)	65.0 (10.0)	0.619
sPAP (mmHg), median (IQR)	39.0 (24.8)	36.0 (30.0)	30.0 (11.0)	**< 0.001**
LV diastolic dysfunction, (%)	48.1	55.9	40.3	0.222
Pulmonary hypertension, (%)	53.6	48.6	17.9	**< 0.001**
ANA, yes (%)	100.0	94.3	98.1	0.269
ACA, yes (%)	17.2	31.4	30.1	0.342
Scl-70, yes (%)	58.6	45.7	42.3	0.267
ESR elevation, (%)	55.2	38.2	29.0	**0.020**
CRP elevation, (%)	37.9	38.2	28.6	0.390
Immunosuppressants, (%)	86.2	82.9	70.9	0.137
Corticosteroids, (%)	82.8	60.0	52.6	**0.010**
Vasoactive drugs, (%)	79.3	76.5	52.6	**0.003**

SSc: Systemic sclerosis; PE: Pericardial effusion; S.D.: Standard deviation; IQR: Interquartile range; GI: Gastrointestinal; ILD: interstitial lung disease; FVC: forced vital capacity; DLCO: carbon monoxide diffusing capacity; sPAP: Systolic pulmonary artery pressure, as assessed by echocardiography; LV: Left ventricle; EF: Ejection fraction; ANA: antinuclear antibodies; ACA: anti-centromere antibodies; Scl-70: anti-topoisomerase I antibodies; ESR: Erythrocyte sedimentation rate; CRP: C-reactive protein

**Fig. 2:**
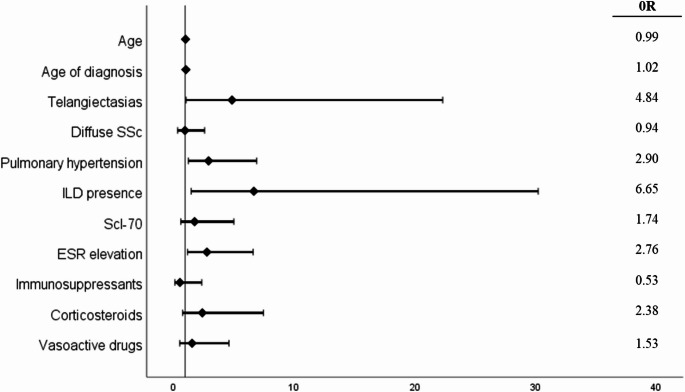
Characteristics associated with persistent pericardial effusion in SSc patients in the multivariate logistic regression. SSc: Systemic sclerosis; ILD: interstitial lung disease; Scl-70: anti-topoisomerase I antibodies; ESR: Erythrocyte sedimentation rate

Univariate and multivariate analyses results for persistent PerEff are summarised in Table S2 (see supplementary data available). In the univariate analysis a significant association was observed with corticosteroid (*p* < 0.01) and vasoactive drug use (*p* < 0.05), elevated ESR (*p* < 0.05), the presence of PH (*p* = 0.001), ILD (*p* < 0.01) and telangiectasias (*p* < 0.05). No association was found between elevated CRP levels and persistent PerEff, therefore only elevated ESR was included in the model. In the multivariate analysis, again, the presence of PH [OR 2.903 (95% CI: 1.222–6.894)], ILD [OR 6.653 (95% CI: 1.465–30.218)], telangiectasias [OR 4.843 (95% CI: 1.051–22.301)] and increased ESR [OR 2.763 (95% CI: 1.157–6.597)] remained significant independent predictors of persistent PerEff (Fig. 2, Table S2). Overall, the model predicted correctly 86.5% of the cases and neither interaction between PH and ILD, nor between PH and vasoactive drugs made a statistically significant contribution to the multivariate model (Table S2, see supplementary data available).

## Discussion

To the extent of our knowledge, this is the first study exploring the presence of PerEff among SSc patients, with special interest on persistent PerEff and trying to identify associated factors. The results revealed that almost one out of four SSc patients (24.5%) had at least one echocardiogram ever positive for PerEff and more than one out of ten SSc patients (13.2%) exhibited persistent PerEff. The majority of the SSc patients, however, remained persistently negative for PerEff. Most importantly, both ever and persistent PerEff were associated with the presence of PH, ILD, telangiectasias and increased ESR in the multivariate analysis.

In more detail, PH was significantly more frequent among individuals with any occurrence PerEff. Likewise, SSc patients without history of PerEff had lower frequency of PH compared to those with single or persistent PerEff. Furthermore, PH remained a significant independent predictor of ever and persistent PerEff. In accordance with our results, a recent study from the PHAROS registry, showed that half of SSc-PH patients had at least one echocardiogram positive for PerEff at or after PH diagnosis. Moreover, in the same cohort, persistent PerEff was associated with poorer prognosis, underlying its clinical importance [14]. Furthermore, SSc patients with history of PerEff exhibited increased ILD prevalence, lower FVC and lower DLCO values as compared to those who never had PerEff. To this end, SSc patients with never PerEff had lower frequency of ILD, as well as higher FVC and DLCO values compared to patients with single and persistent PerEff. Although pericardial abnormalities are commonly seen among SSc patients with ILD, the potential causative relationship remains unclear [34].

In our study telangiectasias were, independently, associated with both ever and persistent PerEff. In fact, telangiectasias have, also, been associated with an increased risk for the development of SSc-related primary heart involvement in the European Scleroderma Trials and Research Group (EUSTAR) cohort, supporting the microvascular hypothesis for heart involvement in SSc [12]. Likewise, our data showed that elevated ESR was associated with both ever and persistent PerEff in the multivariate analysis. Increased inflammatory markers are non-specific markers of disease activity in SSc [35]. Indeed, ESR and CRP have emerged as risk factors for ILD in connective tissue diseases, including SSc [36]. Moreover, elevated ESR has, also, been associated with PH, low FVC, low DLCO and has been integrated in various 5-year prognostic models for SSc [37–39]. ESR and CRP are, also, suggested annually as a nonspecific workup that could indicate cardiac disease [40].

In our cohort, individuals with ever PerEff had older age, along with older age of SSc diagnosis. In a French cohort study by Guédon et al., patients with SSc-related cardiac disease were older than those without any cardiac disease [9]. Additionally, age increased the risk for having heart involvement in the EUSTAR cohort [12]. Furthermore, GI involvement was more common in our patients with ever PerEff. Intestinal symptoms were recently reported to increase the risk for the development of SSc-related primary heart involvement in the EUSTAR cohort [12]. In previous studies, diffuse skin subtype had, also, emerged as a risk factor for pericardial involvement [9, 25]. In our analysis, however, neither diffuse SSc subtype, nor the presence of anti-Scl-70 were significantly associated with ever or persistent PerEff. This could be, at least partly, attributed to the differences between populations.

In the present cohort, no statistically significant differences between the groups were observed regarding the use of immunosuppressants. Corticosteroids and vasoactive drugs were significantly less frequently used by individuals that never had PerEff compared to patients with both single and persistent PerEff, yet they lost their significance in the multivariate analysis. Currently, there is no consensus available for the treatment of pericardial involvement in SSc and current practice guidelines refer mainly to myocardial inflammation and fibrosis [41, 42]. Non-steroidal anti-inflammatory drugs and colchicine can be used as a first line of treatment in cases of symptomatic pericarditis [8, 41, 43]. Corticosteroids should be used with caution and in refractory cases [8]. This is due to the increased risk for scleroderma renal crisis and because, unlike other autoimmune rheumatic diseases, SSc-related pericarditis may have a blunted response to corticosteroids, possibly related to its pathophysiology [8, 41]. Immunosuppressants (azathioprine, mycophenolate mofetil and cyclophosphamide) have also been used successfully in some cases [8, 44]. Pericardial drainage, although recommended in patients with large PerEff or tamponade, has been associated with poor outcomes, especially when it is PH-associated [8, 43]. Currently, there is an absence of therapeutic options targeting specifically cardiac fibrosis, although recent data may support the use of antifibrotic drugs. In a small study, a positive impact of antifibrotic treatment on SSc primary heart involvement was detected. In more details, there was a significant improvement in myocardial extracellular volume (which correlates with myocardial histology) in the nintedanib group compared to the control group, as measured by cardiac magnetic resonance at 6 months. Additionally, the modification in right ventricular ejection fraction was significantly different between groups, favouring the nintedanib group [45, 46].

Heart involvement in SSc has been reported as one of the main risk factors for poor outcome [5]. It should be considered particularly in the early stages of the disease, albeit it may also develop throughout the disease course [40, 42]. Clinically manifest heart involvement is associated with a 5-year mortality of 75% [12]. Therefore, screening for heart involvement should be performed in every SSc patient at the time of diagnosis [40]. Asymptomatic SSc patients without heart involvement (that can be stratified into lower and higher risk) should have an annual assessment [40, 42]. For symptomatic patients or patients diagnosed with heart involvement, the frequency of monitoring should be tailored by a multidisciplinary team [40, 42]. In general, according to the European Society of Cardiology, the follow-up of pericardial effusion is mainly based on the evaluation of symptoms and the echocardiographic size of the effusion, as well as additional features such as inflammatory markers (e.g. CRP) [21].

The underlying mechanisms of PerEff in SSc are not fully understood. A significant independent association with PH was reported in the present study. The prevalence of PerEff in PH is increased, especially in patients with autoimmune rheumatic diseases [14, 26, 47]. One of the leading hypotheses suggests an impaired pericardial fluid drainage into the right atrium. In fact, mean right atrial pressure is the most closely related to the size of pericardial fluid. Consequently, an increased right atrial pressure intervenes in fluid reabsorption, resulting in its accumulation [26, 47]. On the contrary inflammatory conditions have been proposed to contribute independently to the development of PerEff [26]. Nonetheless, pathogenesis of PerEff in SSc is considered to be distinct, compared to other autoimmune rheumatic diseases [8, 16, 41]. This is because pericardial fluid seems to have characteristics of an exudate, while there is lack of autoantibodies, immunocomplexes or complement consumption [8, 16, 41, 48, 49]. At histological examination, there is evidence of chronic inflammatory infiltrates and pericardial fibrosis [8, 15]. Neutrophilic infiltrates and granulomas have, also, been described [15, 49]. In support of the above, in our cohort PerEff (ever and persistent) was associated with the presence of both increased inflammatory markers and ILD.

Pericardial involvement is common among patients with autoimmune rheumatic diseases, including SSc. Most studies in SSc, focus on the possible association between pericardial effusion and mortality. The present study tries to elucidate factors that could be associated with PerEff in a cohort of SSc patients. Despite the interesting findings of our study, there are some unavoidable limitations. First of all, the retrospective design of the study that could lead to certain forms of bias. Furthermore, the fact that health data were tracked over an extended period, affected the available information on the methodology of the echocardiograms performed. In addition, due to inadequacy of data, regarding the volume and sometimes the semiquantitative classification of pericardial fluid, no further analyses could be performed. Another limitation could be the lack of detailed information regarding symptoms on presentation of the patients with PerEff, which however was beyond the scope of this article. Although skin involvement (diffuse or limited) was evaluated in the present study, no outcome measure for skin thickness was used in the analysis (such as the modified Rodnan’s skin score, mRSS) mainly due to lack of data before mRSS became widely accepted [50]. In addition, all the participants were of European ancestry, making it difficult to generalise the results.

## Conclusion

In conclusion, we have showed that PH, ILD, telangiectasias and increased inflammatory burden were associated with single and persistent PerEff. Moreover, PerEff was a quite common SSc-related manifestation. The above-mentioned results should be taken into consideration in clinical routine, also keeping in mind the poorer outcomes that have been related to PerEff in SSc individuals. Therefore, it is crucial to identify asymptomatic patients at higher risk of developing heart involvement, which remains an important unmet need. This is particularly important as heart involvement usually presents in the early stages of the disease, although it can develop throughout the disease course. However, larger and prospective studies are needed to confirm these results, identify other risk factors, investigate possible underlying mechanisms, evaluate potential treatment options (both immunosuppressives and antifibrotics) and help develop clinical practice guidelines.

## Supplementary Information

Below is the link to the electronic supplementary material.

Supplementary Material 1

## Data Availability

The authors will make available participant anonymised data on reasonable request. Data requests should be directed to the corresponding author.
