# Supplementary material for: Factors associated with pericardial effusion development and persistence in systemic sclerosis: a single-centre retrospective analysis
Source: Rheumatol Int. 2025 Jul 14;45(8):169. doi: 10.1007/s00296-025-05925-0 (PMC12259800; doi:10.1007/s00296-025-05925-0)
Supplement: Supplementary file 1 — Supplementary Material 1 [file 296_2025_5925_MOESM1_ESM.docx]

Supplementary material

Supplementary table S1

| Table S1. Univariate and multivariate logistic regression analysis for ever PE in SSc patients | | | | | |
| --- | --- | --- | --- | --- | --- |
|  | Univariate Model | | Multivariate Model | | |
| Variables | OR | P value | 0R | 95% CI | P value |
| Age (years) | 1.029 | **0.007** | 0.994 | (0.951 – 1.038) | 0.772 |
| Age of diagnosis (years) | 1.019 | **0.048** | 1.021 | (0.984 – 1.060) | 0.269 |
| Male sex | 0.646 | 0.294 |  |  |  |
| Telangiectasias | 3.419 | **0.001** | 4.245 | (1.624 – 11.095) | **0.003** |
| GI involvement | 1.838 | **0.043** | 1.890 | (0.809 – 4.416) | 0.142 |
| Diffuse SSc | 1.604 | 0.103 | 0.819 | (0.367 – 1.826) | 0.626 |
| LV diastolic dysfunction | 1.669 | 0.073 | 1.084 | (0.512 – 2.297) | 0.833 |
| PH | 4.919 | **0.001** | 3.219 | (1.612 – 6.430) | **0.001** |
| ILD presence | 3.180 | **0.001** | 4.184 | (1.765 – 9.917) | **0.001** |
| Scl-70 | 1.659 | 0.067 | 1.677 | (0.782 – 3.596) | 0.184 |
| ESR elevation | 2.282 | **0.004** | 2.013 | (1.014 – 3.997) | **0.046** |
| Vasoactive drugs | 3.107 | **0.001** | 1.885 | (0.886 – 4.011) | 0.100 |
| Corticosteroids | 1.705 | 0.062 | 0.722 | (0.327 – 1.593) | 0.420 |
| PH*ILD | 6.118 | **0.001** | 1.672 | (0.247 – 11.299) | 0.598 |
| PH*Vasoactive drugs | 6.201 | **0.001** | 0.949 | (0.168 – 5.346) | 0.952 |

SSc: Systemic sclerosis; PE: Pericardial effusion; PH: Pulmonary hypertension; ILD: Interstitial lung disease; LV: Left ventricle; GI: Gastrointestinal; Scl-70: Anti-topoisomerase I antibodies; ESR: Erythrocyte sedimentation rate

| Supplementary table S2  Table S2. Univariate and multivariate logistic regression analysis for persistent PE in SSc patients | | | | | |
| --- | --- | --- | --- | --- | --- |
|  | Univariate Model | | Multivariate Model | | |
| Variables | OR | P value | 0R | 95% CI | P value |
| Age (years) | 1.022 | 0.158 | 0.992 | (0.936 – 1.051) | 0.778 |
| Age of diagnosis (years) | 1.020 | 0.156 | 1.022 | (0.972 – 1.075) | 0.394 |
| Male sex | 0.619 | 0.455 |  |  |  |
| Telangiectasias | 6.539 | **0.012** | 4.843 | (1.051 – 22.301) | **0.043** |
| GI involvement | 1.698 | 0.249 |  |  |  |
| Diffuse SSc | 1.910 | 0.111 | 0.943 | (0.346 – 2.570) | 0.909 |
| LV diastolic dysfunction | 1.227 | 0.620 |  |  |  |
| PH | 3.744 | **0.001** | 2.903 | (1.222 – 6.894) | **0.016** |
| ILD presence | 7.463 | **0.007** | 6.653 | (1.465 – 30.218) | **0.014** |
| Scl-70 | 1.883 | 0.118 | 1.742 | (0.607 – 4.994) | 0.302 |
| ESR elevation | 2.780 | **0.012** | 2.763 | (1.157 – 6.597) | **0.022** |
| Immunosuppressants | 2.216 | 0.158 | 0.526 | (0.118 – 2.342) | 0.399 |
| Vasoactive drugs | 2.910 | **0.026** | 1.532 | (0.511 – 4.591) | 0.446 |
| Corticosteroids | 4.094 | **0.006** | 2.381 | (0.760 – 7.456) | 0.136 |
| PH*ILD | 4.969 | **0.001** | 1.248 | (0.053 – 29.276) | 0.891 |
| PH*Vasoactive drugs | 4.758 | **0.001** | 1.819 | (0.451 – 7.332) | 0.400 |

SSc: Systemic sclerosis; PE: Pericardial effusion; PH: Pulmonary hypertension; ILD: Interstitial lung disease; LV: Left ventricle; GI: Gastrointestinal; Scl-70: Anti-topoisomerase I antibodies; ESR: erythrocyte sedimentation rate
